# Military Surgeons and Combatant Officers

**Published:** 1904-08-27

**Authors:** 


### Military Surgeons and Combatant Officers.

For some time past it has been felt in the United States of America that the most desirable class of candidates was not being attracted into the Medical Corps of the Army, and the recently amended regulations for admission into that service are the outcome of a desire for improvement in this respect. Henceforward all candidates will be subjected to a preliminary examination, and then, if successful, to a final or qualifying one, with an intervening course of instruction at the Army Medical School at Washington. The preliminary examination will consist of a rigid inquiry into the physical qualifications of those seeking commissions and a written examination in mathematics, geography, Latin grammar and the reading of easy Latin prose, English grammar, orthography and composition, anatomy, physiology, chemistry and physics, materia medica and therapeutics, and normal histology. The preliminary examinations will be conducted simultaneously at several centres throughout the United States, and all the candidates' papers will be forwarded for criticism and marking to Washington. While there is little doubt that the new regulations will serve to raise the standard of education, and consequently to improve the social condition of the United States Army Medical Service, it must of necessity, as the *New York Medical News* points out, fail to remove the frequently manifested antagonism between the medical and combatant officers of the Army. This latter problem is one which arises in our own military service, and in most countries where the Army medical departments are officered by men of the same social origin as the combatant branches, and the cause does not lie on the surface. It must be looked for in the totally different traditions which rule the two professions of the soldier and the doctor; and so long as this fact is ignored no amount of regulations for more exacting examinations or increased pay will overcome the difficulty. The solution must be worked out from within, and the onus lies with each individual army surgeon and combatant officer.

The desire for popular applause is a primitive instinct of man, and its realisation belongs to the soldier by right, sanctioned by tradition throughout the ages. But medicine, tracing its ancestry back to priests and religious bodies, regards the glamour of public recognition as being without its sphere, and its follower must regard such ambition as forbidden fruit to which he may not aspire without the hazard of being untrue to himself and his profession. The calling of medicine has its own hardships and its own dangers, which, spread over a life, are probably greater than those of a soldier's career; but there is this difference that, in the one case they must for the most part be endured alone with only self-respect for their assured recompense, while the soldier may look to material reward, to honours, to public adulation.

The fact that army surgeons are sometimes impatient for military titles and status is an indication that a certain proportion of those who have entered the Army Medical Service have mistaken their calling, for they lack the moral courage to resist the attractions of a soldier's life, and to patiently watch from a nobler standpoint the little foibles which beset their fellow-men. There was an army surgeon who was once reprimanded for not wearing his medals—received for service in the Crimea, the Indian Mutiny, and other fields. "These are the decorations of a soldier," he replied, "to which, being a doctor, I have no claim." And there was no man more respected and loved by his soldier comrades than this surgeon, whose every action in life showed that modesty and patience which are the very essence of the true spirit of medicine.

